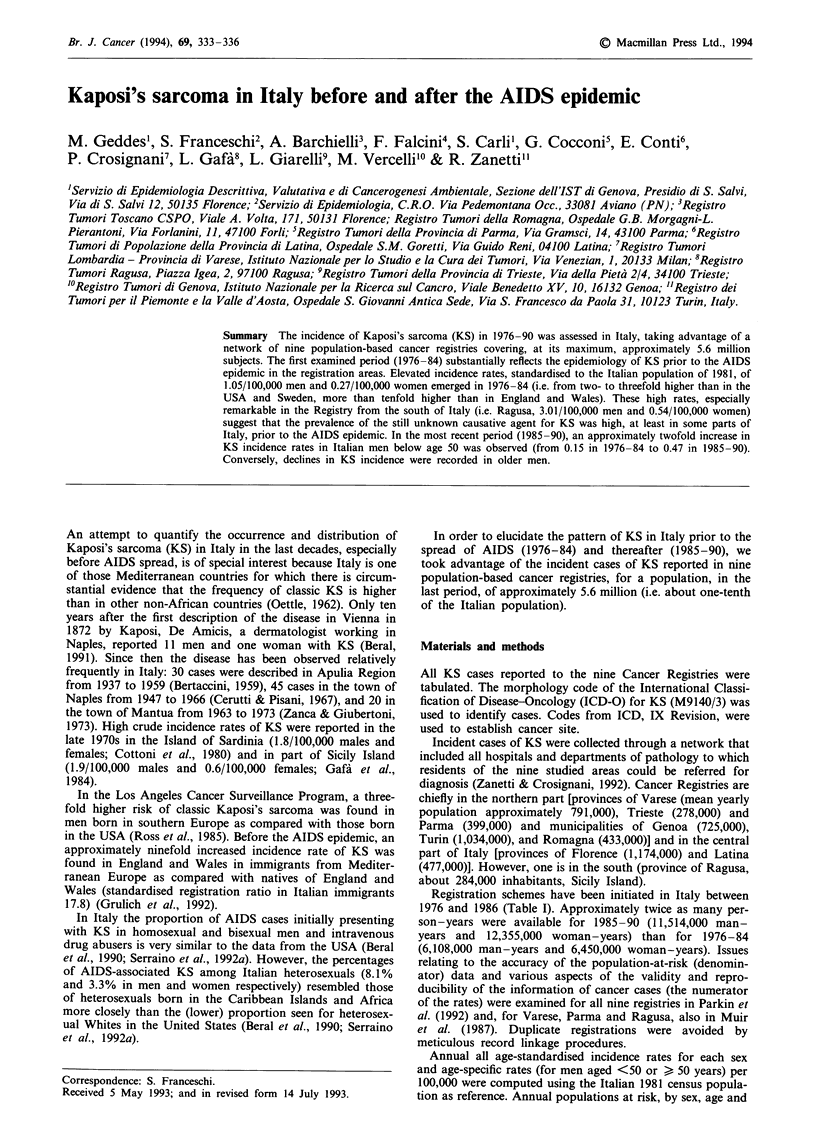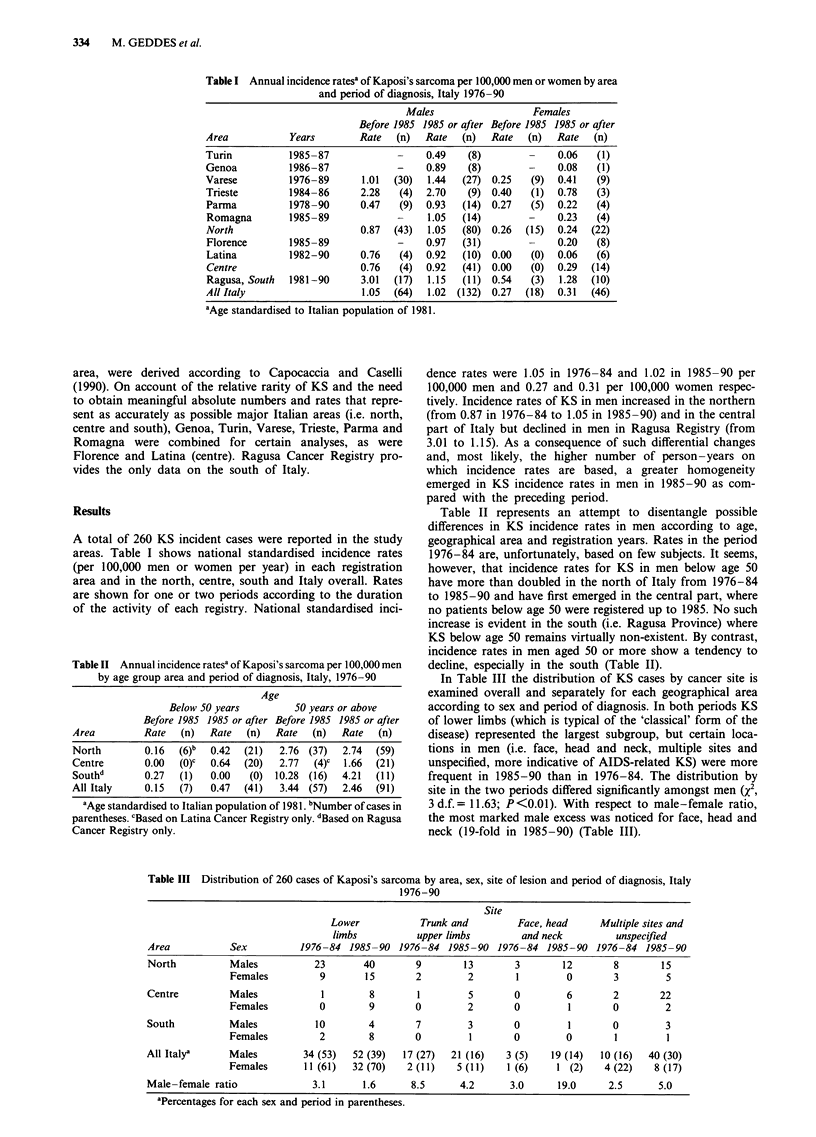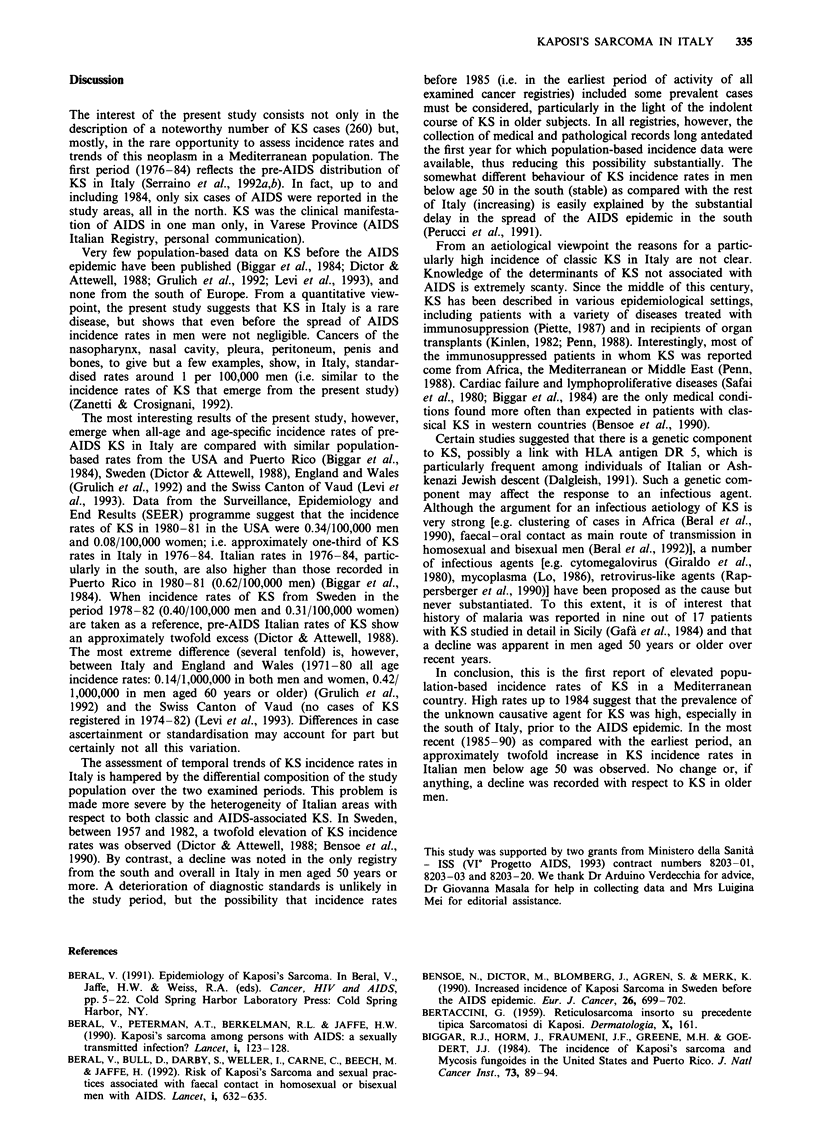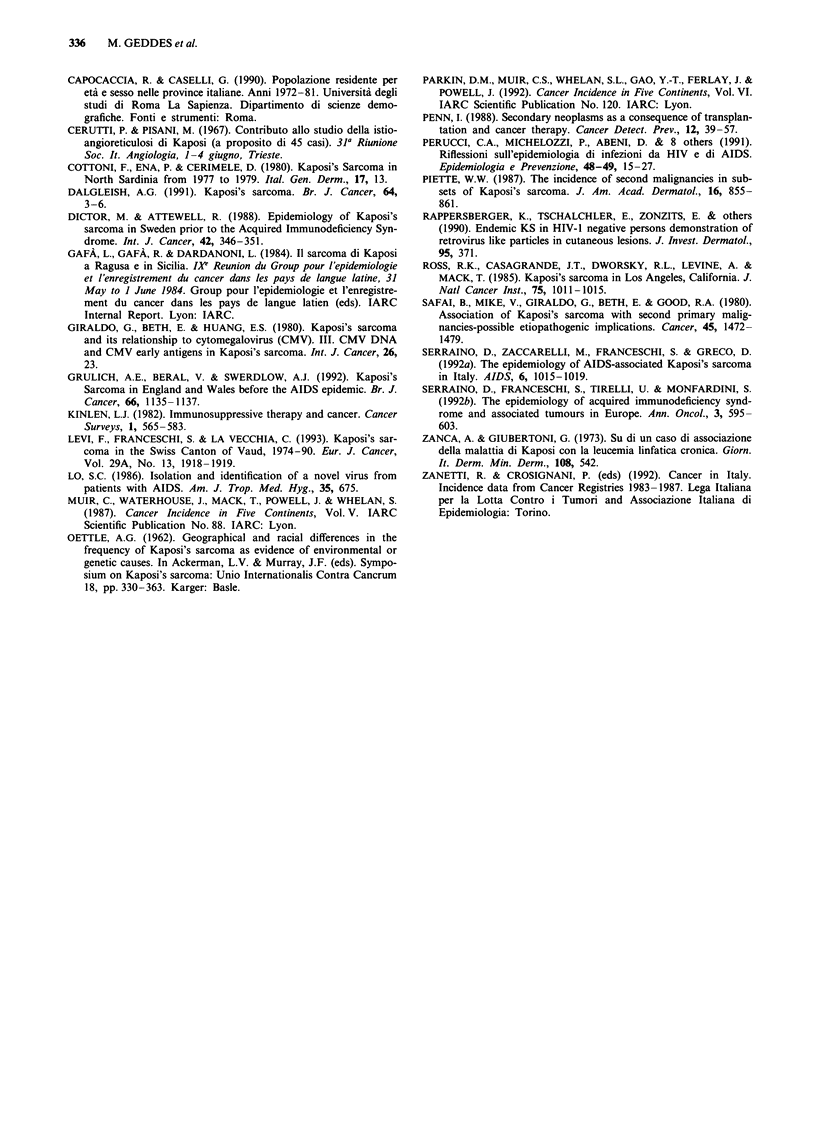# Kaposi's sarcoma in Italy before and after the AIDS epidemic.

**DOI:** 10.1038/bjc.1994.60

**Published:** 1994-02

**Authors:** M. Geddes, S. Franceschi, A. Barchielli, F. Falcini, S. Carli, G. Cocconi, E. Conti, P. Crosignani, L. Gafà, L. Giarelli

**Affiliations:** Servizio di Epidemiologia Descrittiva, Valutativa e di Cancerogenesi Ambientale, Sezione dell'IST di Genova, Italy.

## Abstract

The incidence of Kaposi's sarcoma (KS) in 1976-90 was assessed in Italy, taking advantage of a network of nine population-based cancer registries covering, at its maximum, approximately 5.6 million subjects. The first examined period (1976-84) substantially reflects the epidemiology of KS prior to the AIDS epidemic in the registration areas. Elevated incidence rates, standardised to the Italian population of 1981, of 1.05/100,000 men and 0.27/100,000 women emerged in 1976-84 (i.e. from two- to threefold higher than in the USA and Sweden, more than tenfold higher than in England and Wales). These high rates, especially remarkable in the Registry from the south of Italy (i.e. Ragusa, 3.01/100,000 men and 0.54/100,000 women) suggest that the prevalence of the still unknown causative agent for KS was high, at least in some parts of Italy, prior to the AIDS epidemic. In the most recent period (1985-90), an approximately twofold increase in KS incidence rates in Italian men below age 50 was observed (from 0.15 in 1976-84 to 0.47 in 1985-90). Conversely, declines in KS incidence were recorded in older men.


Br. J. Cancer (1994), 69, 333-336 © Macmillan Press Ltd., 1994

### Kaposi's sarcoma in Italy before and after the AIDS epidemic

M. Geddes[1], S. Franceschi[2], A. Barchielli[3], F. Falcini[4], S. Carli[1], G. Cocconi[5], E. Conti[6], P. Crosignani[7], L. Gafà[8], L. Giarelli[9], M. Vercelli[10] & R. Zanetti[11]

[1]*Servizio di Epidemiologia Descrittiva, Valutativa e di Cancerogenesi Ambientale, Sezione dell'IST di Genova, Presidio di S. Salvi, Via di S. Salvi 12, 50135 Florence;* [2]*Servizio di Epidemiologia, C.R.O. Via Pedemontana Occ., 33081 Aviano (PN);* [3]*Registro Tumori Toscano CSPO, Viale A. Volta, 171, 50131 Florence; Registro Tumori della Romagna, Ospedale G.B. Morgagni-L. Pierantoni, Via Forlanini, 11, 47100 Forlì;* [5]*Registro Tumori della Provincia di Parma, Via Gramsci, 14, 43100 Parma;* [6]*Registro Tumori di Popolazione della Provincia di Latina, Ospedale S.M. Goretti, Via Guido Reni, 04100 Latina;* [7]*Registro Tumori Lombardia – Provincia di Varese, Istituto Nazionale per lo Studio e la Cura dei Tumori, Via Venezian, 1, 20133 Milan;* [8]*Registro Tumori Ragusa, Piazza Igea, 2, 97100 Ragusa;* [9]*Registro Tumori della Provincia di Trieste, Via della Pietà 2/4, 34100 Trieste;* [10]*Registro Tumori di Genova, Istituto Nazionale per la Ricerca sul Cancro, Viale Benedetto XV, 10, 16132 Genoa;* [11]*Registro dei Tumori per il Piemonte e la Valle d'Aosta, Ospedale S. Giovanni Antica Sede, Via S. Francesco da Paola 31, 10123 Turin, Italy.*

**Summary** The incidence of Kaposi's sarcoma (KS) in 1976–90 was assessed in Italy, taking advantage of a network of nine population-based cancer registries covering, at its maximum, approximately 5.6 million subjects. The first examined period (1976–84) substantially reflects the epidemiology of KS prior to the AIDS epidemic in the registration areas. Elevated incidence rates, standardised to the Italian population of 1981, of 1.05/100,000 men and 0.27/100,000 women emerged in 1976–84 (i.e. from two- to threefold higher than in the USA and Sweden, more than tenfold higher than in England and Wales). These high rates, especially remarkable in the Registry from the south of Italy (i.e. Ragusa, 3.01/100,000 men and 0.54/100,000 women) suggest that the prevalence of the still unknown causative agent for KS was high, at least in some parts of Italy, prior to the AIDS epidemic. In the most recent period (1985–90), an approximately twofold increase in KS incidence rates in Italian men below age 50 was observed (from 0.15 in 1976–84 to 0.47 in 1985–90). Conversely, declines in KS incidence were recorded in older men.

An attempt to quantify the occurrence and distribution of Kaposi's sarcoma (KS) in Italy in the last decades, especially before AIDS spread, is of special interest because Italy is one of those Mediterranean countries for which there is circumstantial evidence that the frequency of classic KS is higher than in other non-African countries (Oettle, 1962). Only ten years after the first description of the disease in Vienna in 1872 by Kaposi, De Amicis, a dermatologist working in Naples, reported 11 men and one woman with KS (Beral, 1991). Since then the disease has been observed relatively frequently in Italy: 30 cases were described in Apulia Region from 1937 to 1959 (Bertaccini, 1959), 45 cases in the town of Naples from 1947 to 1966 (Cerutti & Pisani, 1967), and 20 in the town of Mantua from 1963 to 1973 (Zanca & Giubertoni, 1973). High crude incidence rates of KS were reported in the late 1970s in the Island of Sardinia (1.8/100,000 males and females; Cottoni *et al.*, 1980) and in part of Sicily Island (1.9/100,000 males and 0.6/100,000 females; Gafà *et al.*, 1984).

In the Los Angeles Cancer Surveillance Program, a threefold higher risk of classic Kaposi's sarcoma was found in men born in southern Europe as compared with those born in the USA (Ross *et al.*, 1985). Before the AIDS epidemic, an approximately ninefold increased incidence rate of KS was found in England and Wales in immigrants from Mediterranean Europe as compared with natives of England and Wales (standardised registration ratio in Italian immigrants 17.8) (Grulich *et al.*, 1992).

In Italy the proportion of AIDS cases initially presenting with KS in homosexual and bisexual men and intravenous drug abusers is very similar to the data from the USA (Beral *et al.*, 1990; Serraino *et al.*, 1992*a*). However, the percentages of AIDS-associated KS among Italian heterosexuals (8.1% and 3.3% in men and women respectively) resembled those of heterosexuals born in the Caribbean Islands and Africa more closely than the (lower) proportion seen for heterosexual Whites in the United States (Beral *et al.*, 1990; Serraino *et al.*, 1992*a*).

Correspondence: S. Franceschi.
Received 5 May 1993; and in revised form 14 July 1993.

In order to elucidate the pattern of KS in Italy prior to the spread of AIDS (1976–84) and thereafter (1985–90), we took advantage of the incident cases of KS reported in nine population-based cancer registries, for a population, in the last period, of approximately 5.6 million (i.e. about one-tenth of the Italian population).

### Materials and methods

All KS cases reported to the nine Cancer Registries were tabulated. The morphology code of the International Classification of Disease–Oncology (ICD-O) for KS (M9140/3) was used to identify cases. Codes from ICD, IX Revision, were used to establish cancer site.

Incident cases of KS were collected through a network that included all hospitals and departments of pathology to which residents of the nine studied areas could be referred for diagnosis (Zanetti & Crosignani, 1992). Cancer Registries are chiefly in the northern part [provinces of Varese (mean yearly population approximately 791,000), Trieste (278,000) and Parma (399,000) and municipalities of Genoa (725,000), Turin (1,034,000), and Romagna (433,000)] and in the central part of Italy [provinces of Florence (1,174,000) and Latina (477,000)]. However, one is in the south (province of Ragusa, about 284,000 inhabitants, Sicily Island).

Registration schemes have been initiated in Italy between 1976 and 1986 (Table I). Approximately twice as many person–years were available for 1985–90 (11,514,000 man–years and 12,355,000 woman–years) than for 1976–84 (6,108,000 man–years and 6,450,000 woman–years). Issues relating to the accuracy of the population-at-risk (denominator) data and various aspects of the validity and reproducibility of the information of cancer cases (the numerator of the rates) were examined for all nine registries in Parkin *et al.* (1992) and, for Varese, Parma and Ragusa, also in Muir *et al.* (1987). Duplicate registrations were avoided by meticulous record linkage procedures.

Annual all age-standardised incidence rates for each sex and age-specific rates (for men aged <50 or ⩾ 50 years) per 100,000 were computed using the Italian 1981 census population as reference. Annual populations at risk, by sex, age and

**Table I** Annual incidence rates[a] of Kaposi's sarcoma per 100,000 men or women by area and period of diagnosis, Italy 1976–90

| | | Males | | | | Females | | | |
|---|---|---|---|---|---|---|---|---|---|
| | | Before 1985 | | 1985 or after | | Before 1985 | | 1985 or after | |
| Area | Years | Rate | (n) | Rate | (n) | Rate | (n) | Rate | (n) |
| Turin | 1985–87 | | – | 0.49 | (8) | | – | 0.06 | (1) |
| Genoa | 1986–87 | | – | 0.89 | (8) | | – | 0.08 | (1) |
| Varese | 1976–89 | 1.01 | (30) | 1.44 | (27) | 0.25 | (9) | 0.41 | (9) |
| Trieste | 1984–86 | 2.28 | (4) | 2.70 | (9) | 0.40 | (1) | 0.78 | (3) |
| Parma | 1978–90 | 0.47 | (9) | 0.93 | (14) | 0.27 | (5) | 0.22 | (4) |
| Romagna | 1985–89 | | – | 1.05 | (14) | | – | 0.23 | (4) |
| *North* | | 0.87 | (43) | 1.05 | (80) | 0.26 | (15) | 0.24 | (22) |
| Florence | 1985–89 | | – | 0.97 | (31) | | – | 0.20 | (8) |
| Latina | 1982–90 | 0.76 | (4) | 0.92 | (10) | 0.00 | (0) | 0.06 | (6) |
| *Centre* | | 0.76 | (4) | 0.92 | (41) | 0.00 | (0) | 0.29 | (14) |
| Ragusa, *South* | 1981–90 | 3.01 | (17) | 1.15 | (11) | 0.54 | (3) | 1.28 | (10) |
| *All Italy* | | 1.05 | (64) | 1.02 | (132) | 0.27 | (18) | 0.31 | (46) |

[a]Age standardised to Italian population of 1981.

area, were derived according to Capocaccia and Caselli (1990). On account of the relative rarity of KS and the need to obtain meaningful absolute numbers and rates that represent as accurately as possible major Italian areas (i.e. north, centre and south), Genoa, Turin, Varese, Trieste, Parma and Romagna were combined for certain analyses, as were Florence and Latina (centre). Ragusa Cancer Registry provides the only data on the south of Italy.

### Results

A total of 260 KS incident cases were reported in the study areas. Table I shows national standardised incidence rates (per 100,000 men or women per year) in each registration area and in the north, centre, south and Italy overall. Rates are shown for one or two periods according to the duration of the activity of each registry. National standardised incidence rates were 1.05 in 1976–84 and 1.02 in 1985–90 per 100,000 men and 0.27 and 0.31 per 100,000 women respectively. Incidence rates of KS in men increased in the northern (from 0.87 in 1976–84 to 1.05 in 1985–90) and in the central part of Italy but declined in men in Ragusa Registry (from 3.01 to 1.15). As a consequence of such differential changes and, most likely, the higher number of person-years on which incidence rates are based, a greater homogeneity emerged in KS incidence rates in men in 1985–90 as compared with the preceding period.

**Table II** Annual incidence rates[a] of Kaposi's sarcoma per 100,000 men by age group area and period of diagnosis, Italy, 1976–90

| | Age | | | | | | | |
|---|---|---|---|---|---|---|---|---|
| | Below 50 years | | | | 50 years or above | | | |
| | Before 1985 | | 1985 or after | | Before 1985 | | 1985 or after | |
| Area | Rate | (n) | Rate | (n) | Rate | (n) | Rate | (n) |
| North | 0.16 | (6)[b] | 0.42 | (21) | 2.76 | (37) | 2.74 | (59) |
| Centre | 0.00 | (0)[c] | 0.64 | (20) | 2.77 | (4)[c] | 1.66 | (21) |
| South[d] | 0.27 | (1) | 0.00 | (0) | 10.28 | (16) | 4.21 | (11) |
| All Italy | 0.15 | (7) | 0.47 | (41) | 3.44 | (57) | 2.46 | (91) |

[a]Age standardised to Italian population of 1981. [b]Number of cases in parentheses. [c]Based on Latina Cancer Registry only. [d]Based on Ragusa Cancer Registry only.

Table II represents an attempt to disentangle possible differences in KS incidence rates in men according to age, geographical area and registration years. Rates in the period 1976–84 are, unfortunately, based on few subjects. It seems, however, that incidence rates for KS in men below age 50 have more than doubled in the north of Italy from 1976–84 to 1985–90 and have first emerged in the central part, where no patients below age 50 were registered up to 1985. No such increase is evident in the south (i.e. Ragusa Province) where KS below age 50 remains virtually non-existent. By contrast, incidence rates in men aged 50 or more show a tendency to decline, especially in the south (Table II).

In Table III the distribution of KS cases by cancer site is examined overall and separately for each geographical area according to sex and period of diagnosis. In both periods KS of lower limbs (which is typical of the 'classical' form of the disease) represented the largest subgroup, but certain locations in men (i.e. face, head and neck, multiple sites and unspecified, more indicative of AIDS-related KS) were more frequent in 1985–90 than in 1976–84. The distribution by site in the two periods differed significantly amongst men ($\chi^2$, 3 d.f. = 11.63; $P<0.01$). With respect to male-female ratio, the most marked male excess was noticed for face, head and neck (19-fold in 1985–90) (Table III).

**Table III** Distribution of 260 cases of Kaposi's sarcoma by area, sex, site of lesion and period of diagnosis, Italy 1976–90

| | | Site | | | | | | | |
|---|---|---|---|---|---|---|---|---|---|
| | | Lower limbs | | Trunk and upper limbs | | Face, head and neck | | Multiple sites and unspecified | |
| Area | Sex | 1976–84 | 1985–90 | 1976–84 | 1985–90 | 1976–84 | 1985–90 | 1976–84 | 1985–90 |
| North | Males | 23 | 40 | 9 | 13 | 3 | 12 | 8 | 15 |
| | Females | 9 | 15 | 2 | 2 | 1 | 0 | 3 | 5 |
| Centre | Males | 1 | 8 | 1 | 5 | 0 | 6 | 2 | 22 |
| | Females | 0 | 9 | 0 | 2 | 0 | 1 | 0 | 2 |
| South | Males | 10 | 4 | 7 | 3 | 0 | 1 | 0 | 3 |
| | Females | 2 | 8 | 0 | 1 | 0 | 0 | 1 | 1 |
| All Italy[a] | Males | 34 (53) | 52 (39) | 17 (27) | 21 (16) | 3 (5) | 19 (14) | 10 (16) | 40 (30) |
| | Females | 11 (61) | 32 (70) | 2 (11) | 5 (11) | 1 (6) | 1 (2) | 4 (22) | 8 (17) |
| Male–female ratio | | 3.1 | 1.6 | 8.5 | 4.2 | 3.0 | 19.0 | 2.5 | 5.0 |

[a]Percentages for each sex and period in parentheses.

### Discussion

The interest of the present study consists not only in the description of a noteworthy number of KS cases (260) but, mostly, in the rare opportunity to assess incidence rates and trends of this neoplasm in a Mediterranean population. The first period (1976–84) reflects the pre-AIDS distribution of KS in Italy (Serraino *et al.*, 1992*a,b*). In fact, up to and including 1984, only six cases of AIDS were reported in the study areas, all in the north. KS was the clinical manifestation of AIDS in one man only, in Varese Province (AIDS Italian Registry, personal communication).

Very few population-based data on KS before the AIDS epidemic have been published (Biggar *et al.*, 1984; Dictor & Attewell, 1988; Grulich *et al.*, 1992; Levi *et al.*, 1993), and none from the south of Europe. From a quantitative viewpoint, the present study suggests that KS in Italy is a rare disease, but shows that even before the spread of AIDS incidence rates in men were not negligible. Cancers of the nasopharynx, nasal cavity, pleura, peritoneum, penis and bones, to give but a few examples, show, in Italy, standardised rates around 1 per 100,000 men (i.e. similar to the incidence rates of KS that emerge from the present study) (Zanetti & Crosignani, 1992).

The most interesting results of the present study, however, emerge when all-age and age-specific incidence rates of pre-AIDS KS in Italy are compared with similar population-based rates from the USA and Puerto Rico (Biggar *et al.*, 1984), Sweden (Dictor & Attewell, 1988), England and Wales (Grulich *et al.*, 1992) and the Swiss Canton of Vaud (Levi *et al.*, 1993). Data from the Surveillance, Epidemiology and End Results (SEER) programme suggest that the incidence rates of KS in 1980–81 in the USA were 0.34/100,000 men and 0.08/100,000 women; i.e. approximately one-third of KS rates in Italy in 1976–84. Italian rates in 1976–84, particularly in the south, are also higher than those recorded in Puerto Rico in 1980–81 (0.62/100,000 men) (Biggar *et al.*, 1984). When incidence rates of KS from Sweden in the period 1978–82 (0.40/100,000 men and 0.31/100,000 women) are taken as a reference, pre-AIDS Italian rates of KS show an approximately twofold excess (Dictor & Attewell, 1988). The most extreme difference (several tenfold) is, however, between Italy and England and Wales (1971–80 all age incidence rates: 0.14/1,000,000 in both men and women, 0.42/1,000,000 in men aged 60 years or older) (Grulich *et al.*, 1992) and the Swiss Canton of Vaud (no cases of KS registered in 1974–82) (Levi *et al.*, 1993). Differences in case ascertainment or standardisation may account for part but certainly not all this variation.

The assessment of temporal trends of KS incidence rates in Italy is hampered by the differential composition of the study population over the two examined periods. This problem is made more severe by the heterogeneity of Italian areas with respect to both classic and AIDS-associated KS. In Sweden, between 1957 and 1982, a twofold elevation of KS incidence rates was observed (Dictor & Attewell, 1988; Bensoe *et al.*, 1990). By contrast, a decline was noted in the only registry from the south and overall in Italy in men aged 50 years or more. A deterioration of diagnostic standards is unlikely in the study period, but the possibility that incidence rates before 1985 (i.e. in the earliest period of activity of all examined cancer registries) included some prevalent cases must be considered, particularly in the light of the indolent course of KS in older subjects. In all registries, however, the collection of medical and pathological records long antedated the first year for which population-based incidence data were available, thus reducing this possibility substantially. The somewhat different behaviour of KS incidence rates in men below age 50 in the south (stable) as compared with the rest of Italy (increasing) is easily explained by the substantial delay in the spread of the AIDS epidemic in the south (Perucci *et al.*, 1991).

From an aetiological viewpoint the reasons for a particularly high incidence of classic KS in Italy are not clear. Knowledge of the determinants of KS not associated with AIDS is extremely scanty. Since the middle of this century, KS has been described in various epidemiological settings, including patients with a variety of diseases treated with immunosuppression (Piette, 1987) and in recipients of organ transplants (Kinlen, 1982; Penn, 1988). Interestingly, most of the immunosuppressed patients in whom KS was reported come from Africa, the Mediterranean or Middle East (Penn, 1988). Cardiac failure and lymphoproliferative diseases (Safai *et al.*, 1980; Biggar *et al.*, 1984) are the only medical conditions found more often than expected in patients with classical KS in western countries (Bensoe *et al.*, 1990).

Certain studies suggested that there is a genetic component to KS, possibly a link with HLA antigen DR 5, which is particularly frequent among individuals of Italian or Ashkenazi Jewish descent (Dalgleish, 1991). Such a genetic component may affect the response to an infectious agent. Although the argument for an infectious aetiology of KS is very strong [e.g. clustering of cases in Africa (Beral *et al.*, 1990), faecal–oral contact as main route of transmission in homosexual and bisexual men (Beral *et al.*, 1992)], a number of infectious agents [e.g. cytomegalovirus (Giraldo *et al.*, 1980), mycoplasma (Lo, 1986), retrovirus-like agents (Rappersberger *et al.*, 1990)] have been proposed as the cause but never substantiated. To this extent, it is of interest that history of malaria was reported in nine out of 17 patients with KS studied in detail in Sicily (Gafà *et al.*, 1984) and that a decline was apparent in men aged 50 years or older over recent years.

In conclusion, this is the first report of elevated population-based incidence rates of KS in a Mediterranean country. High rates up to 1984 suggest that the prevalence of the unknown causative agent for KS was high, especially in the south of Italy, prior to the AIDS epidemic. In the most recent (1985–90) as compared with the earliest period, an approximately twofold increase in KS incidence rates in Italian men below age 50 was observed. No change or, if anything, a decline was recorded with respect to KS in older men.

This study was supported by two grants from Ministero della Sanità – ISS (VI° Progetto AIDS, 1993) contract numbers 8203–01, 8203–03 and 8203–20. We thank Dr Arduino Verdecchia for advice, Dr Giovanna Masala for help in collecting data and Mrs Luigina Mei for editorial assistance.

### References

BERAL, V. (1991). Epidemiology of Kaposi's Sarcoma. In Beral, V., Jaffe, H.W. & Weiss, R.A. (eds). *Cancer, HIV and AIDS*, pp. 5–22. Cold Spring Harbor Laboratory Press: Cold Spring Harbor, NY.

BERAL, V., PETERMAN, A.T., BERKELMAN, R.L. & JAFFE, H.W. (1990). Kaposi's sarcoma among persons with AIDS: a sexually transmitted infection? *Lancet*, **i**, 123–128.

BERAL, V., BULL, D., DARBY, S., WELLER, I., CARNE, C., BEECH, M. & JAFFE, H. (1992). Risk of Kaposi's Sarcoma and sexual practices associated with faecal contact in homosexual or bisexual men with AIDS. *Lancet*, **i**, 632–635.

BENSOE, N., DICTOR, M., BLOMBERG, J., AGREN, S. & MERK, K. (1990). Increased incidence of Kaposi Sarcoma in Sweden before the AIDS epidemic. *Eur. J. Cancer*, **26**, 699–702.

BERTACCINI, G. (1959). Reticulosarcoma insorto su precedente tipica Sarcomatosi di Kaposi. *Dermatologia*, **X**, 161.

BIGGAR, R.J., HORM, J., FRAUMENI, J.F., GREENE, M.H. & GOEDERT, J.J. (1984). The incidence of Kaposi's sarcoma and Mycosis fungoides in the United States and Puerto Rico. *J. Natl Cancer Inst.*, **73**, 89–94.

CAPOCACCIA, R. & CASELLI, G. (1990). Popolazione residente per età e sesso nelle province italiane. Anni 1972–81. Università degli studi di Roma La Sapienza. Dipartimento di scienze demografiche. Fonti e strumenti: Roma.

CERUTTI, P. & PISANI, M. (1967). Contributo allo studio della istio-angioreticulosi di Kaposi (a proposito di 45 casi). *31ª Riunione Soc. It. Angiologia, 1–4 giugno, Trieste.*

COTTONI, F., ENA, P. & CERIMELE, D. (1980). Kaposi's Sarcoma in North Sardinia from 1977 to 1979. *Ital. Gen. Derm.*, **17,** 13.

DALGLEISH, A.G. (1991). Kaposi's sarcoma. *Br. J. Cancer*, **64,** 3–6.

DICTOR, M. & ATTEWELL, R. (1988). Epidemiology of Kaposi's sarcoma in Sweden prior to the Acquired Immunodeficiency Syndrome. *Int. J. Cancer*, **42,** 346–351.

GAFÀ, L., GAFÀ, R. & DARDANONI, L. (1984). Il sarcoma di Kaposi a Ragusa e in Sicilia. *IXᵉ Reunion du Group pour l'epidemiologie et l'enregistrement du cancer dans les pays de langue latine, 31 May to 1 June 1984.* Group pour l'epidemiologie et l'enregistrement du cancer dans les pays de langue latien (eds). IARC Internal Report. Lyon: IARC.

GIRALDO, G., BETH, E. & HUANG, E.S. (1980). Kaposi's sarcoma and its relationship to cytomegalovirus (CMV). III. CMV DNA and CMV early antigens in Kaposi's sarcoma. *Int. J. Cancer*, **26,** 23.

GRULICH, A.E., BERAL, V. & SWERDLOW, A.J. (1992). Kaposi's Sarcoma in England and Wales before the AIDS epidemic. *Br. J. Cancer*, **66,** 1135–1137.

KINLEN, L.J. (1982). Immunosuppressive therapy and cancer. *Cancer Surveys*, **1,** 565–583.

LEVI, F., FRANCESCHI, S. & LA VECCHIA, C. (1993). Kaposi's sarcoma in the Swiss Canton of Vaud, 1974–90. *Eur. J. Cancer*, Vol. 29A, No. 13, 1918–1919.

LO, S.C. (1986). Isolation and identification of a novel virus from patients with AIDS. *Am. J. Trop. Med. Hyg.*, **35,** 675.

MUIR, C., WATERHOUSE, J., MACK, T., POWELL, J. & WHELAN, S. (1987). *Cancer Incidence in Five Continents*, Vol. V. IARC Scientific Publication No. 88. IARC: Lyon.

OETTLE, A.G. (1962). Geographical and racial differences in the frequency of Kaposi's sarcoma as evidence of environmental or genetic causes. In Ackerman, L.V. & Murray, J.F. (eds). Symposium on Kaposi's sarcoma: Unio Internationalis Contra Cancrum 18, pp. 330–363. Karger: Basle.

PARKIN, D.M., MUIR, C.S., WHELAN, S.L., GAO, Y.-T., FERLAY, J. & POWELL, J. (1992). *Cancer Incidence in Five Continents*, Vol. VI. IARC Scientific Publication No. 120. IARC: Lyon.

PENN, I. (1988). Secondary neoplasms as a consequence of transplantation and cancer therapy. *Cancer Detect. Prev.*, **12,** 39–57.

PERUCCI, C.A., MICHELOZZI, P., ABENI, D. & 8 others (1991). Riflessioni sull'epidemiologia di infezioni da HIV e di AIDS. *Epidemiologia e Prevenzione*, **48–49,** 15–27.

PIETTE, W.W. (1987). The incidence of second malignancies in subsets of Kaposi's sarcoma. *J. Am. Acad. Dermatol.*, **16,** 855–861.

RAPPERSBERGER, K., TSCHALCHLER, E., ZONZITS, E. & others (1990). Endemic KS in HIV-1 negative persons demonstration of retrovirus like particles in cutaneous lesions. *J. Invest. Dermatol.*, **95,** 371.

ROSS, R.K., CASAGRANDE, J.T., DWORSKY, R.L., LEVINE, A. & MACK, T. (1985). Kaposi's sarcoma in Los Angeles, California. *J. Natl Cancer Inst.*, **75,** 1011–1015.

SAFAI, B., MIKE, V., GIRALDO, G., BETH, E. & GOOD, R.A. (1980). Association of Kaposi's sarcoma with second primary malignancies-possible etiopathogenic implications. *Cancer*, **45,** 1472–1479.

SERRAINO, D., ZACCARELLI, M., FRANCESCHI, S. & GRECO, D. (1992*a*). The epidemiology of AIDS-associated Kaposi's sarcoma in Italy. *AIDS*, **6,** 1015–1019.

SERRAINO, D., FRANCESCHI, S., TIRELLI, U. & MONFARDINI, S. (1992*b*). The epidemiology of acquired immunodeficiency syndrome and associated tumours in Europe. *Ann. Oncol.*, **3,** 595–603.

ZANCA, A. & GIUBERTONI, G. (1973). Su di un caso di associazione della malattia di Kaposi con la leucemia linfatica cronica. *Giorn. It. Derm. Min. Derm.*, **108,** 542.

ZANETTI, R. & CROSIGNANI, P. (eds) (1992). Cancer in Italy. Incidence data from Cancer Registries 1983–1987. Lega Italiana per la Lotta Contro i Tumori and Associazione Italiana di Epidemiologia: Torino.